# Supplementary material for: Education, intelligence and Alzheimer’s disease: evidence from a multivariable two-sample Mendelian randomization study
Source: Int J Epidemiol. 2020 Jan 31;49(4):1163–72. doi: 10.1093/ije/dyz280 (PMC7660137; doi:10.1093/ije/dyz280)
Supplement: dyz280_Supplementary_Data [file dyz280_supplementary_data.docx]

**Online Supplement**

**Harmonization procedures**

***Causal effects of educational attainment on intelligence***A total of 162 approximately independent genome-wide significant (p<5x10^-8^) single nucleotide polymorphisms (SNPs) were identified as being associated with years of schooling in a GWAS meta-analysis of 293,723 participants.^1^ SNP-outcome estimates were extracted from the recent Multi-Trait Analysis of Genome-wide association studies of fluid intelligence (n= 248,482). Out of the 162 educational attainment SNPs, a total of 157 were identified in the intelligence GWAS. No SNPs were excluded due to low minor allele frequencies. Nine SNPs were excluded due to being associated with intelligence at the genome-wide significance level. Thus 148 SNPs were available for this analysis.

***Causal effects of intelligence on educational attainment***A total of 194 approximately independent genome-wide significant (p<5x10^-8^) single nucleotide polymorphisms (SNPs) were identified as being associated with fluid intelligence in a recent Multi-Trait Analysis of Genome-wide association studies (n= 248,482). SNP-outcome estimates for all 194 SNPs were extracted from the years of schooling GWAS described above. Five SNPs were excluded due to low minor allele frequencies (<1%). Nine SNPs were excluded due to being associated with education at the genome-wide significance level. Thus 180 SNPs were available for the analysis.

***Total effects of educational attainment and intelligence on risk of AD***Of the 162 educational attainment SNPs, 142 were identified in the AD GWAS. No SNPs were excluded due to low minor allele frequency. Thus, SNP-outcome estimates for all 142 SNPs were extracted from the AD GWAS and were available for the analysis. Of the 194 intelligence SNPs, 189 were identified in the AD GWAS. A further 4 SNPs were excluded for having minor allele frequencies <1%. Thus, 185 SNP-outcome estimates were extracted from the AD GWAS and were available for the analysis.

***Palindromic SNPs***All GWAS studies included in the analyses were coded on the forward strand, thus, no palindromic SNPs were excluded from analyses

***Aligning alleles***

In an MR analysis, the effect of a SNP on exposure and an outcome must be harmonised to be relative to the same allele. SNPs for the exposure were coded so that the effect allele was always the ‘increasing allele’ (i.e. increasing years of schooling or intelligence), and the alleles were harmonized so that the effect on the outcome corresponded to the same allele as the exposure.

**MR-Egger regression details**We compared results from the IVW regression to those obtained with MR-Egger regression,^2^ as the use of multiple alleles in MR analyses increases the potential for pleiotropic effects due to aggregation of invalid genetic instruments.^3^ MR-Egger relaxes the assumption that the effects of genetic variants on the outcome operate entirely via the exposure, by not constraining the intercept term to zero in the inverse variance weighted regression described in the main paper. In this instance, the intercept parameter indicates the overall pleiotropic effect of the SNPs on the outcome (i.e. a direct effect on the outcome, independent of the exposure, which would violate MR assumptions), with a non-zero intercept providing evidence for bias due to pleiotropy. MR-Egger estimates remain consistent only if the magnitude of the gene-exposure associations across all variants are independent of their pleiotropic effects (i.e. the InSIDE assumption).^2^ The beta coefficient (or slope) of MR-Egger provides a causal estimate of the exposure on the outcome, accounting for this level of pleiotropy and assuming that the pleiotropic effect of SNPs on the outcome is not correlated with the instrument strength.^2^ As recommended by Bowden *et al*.^4^ the extent to which pleiotropy is balanced across the set of instruments was visually assessed by plotting the causal effect estimates against their precision, using a funnel plot and checking for asymmetry (Figures E to H below).

**Tables and figures**

**Table S1: List of SNPs used from the education GWAS**

|  |  |  |  |  |  | **Association with years of schooling** | | | **Association with Alzheimer’s disease** | | |
| --- | --- | --- | --- | --- | --- | --- | --- | --- | --- | --- | --- |
| **SNP** | **CHR** | **POS** | **A1** | **A2** | **EAF** | **BETA** | **SE** | **P** | **LOR** | **SE** | **P** |
| rs11643654 | 16 | 5.10E+07 | A | C | 0.5989 | 0.011 | 0.003 | 0.000012 | -0.03 | 0.0174 | 0.0841 |
| rs16845580 | 2 | 1.60E+08 | T | C | 0.6306 | 0.016 | 0.003 | 2.10E-10 | -0.0266 | 0.016 | 0.09728 |
| rs12956009 | 18 | 4.50E+07 | C | T | 0.5392 | 0.014 | 0.002 | 1.80E-08 | 0.0058 | 0.0156 | 0.7077 |
| rs17167170 | 7 | 1.30E+08 | A | G | 0.7873 | 0.017 | 0.003 | 1.60E-08 | -0.0244 | 0.0197 | 0.2156 |
| rs10930008 | 2 | 1.60E+08 | G | A | 0.7183 | 0.012 | 0.003 | 0.000014 | -0.0151 | 0.0188 | 0.423 |
| rs34638686 | 3 | 4.90E+07 | T | C | 0.1026 | 0.025 | 0.004 | 4.50E-09 | -0.0259 | 0.0257 | 0.3142 |
| rs140711597 | 3 | 4.80E+07 | C | G | 0.97575 | 0.051 | 0.01 | 2.30E-07 | - | - | - |
| rs7029201 | 9 | 2.30E+07 | A | G | 0.4235 | 0.025 | 0.003 | 6.10E-23 | -0.0241 | 0.0172 | 0.1602 |
| rs6800916 | 3 | 5.00E+07 | T | A | 0.91418 | 0.023 | 0.005 | 8.20E-07 | - | - | - |
| rs4863692 | 4 | 1.40E+08 | T | G | 0.334 | 0.018 | 0.003 | 3.80E-12 | 0.0085 | 0.0168 | 0.6132 |
| rs152603 | 5 | 1.10E+08 | G | A | 0.6418 | 0.014 | 0.003 | 1.30E-07 | 0.0013 | 0.0166 | 0.9385 |
| rs56081191 | 6 | 9.90E+07 | A | G | 0.07276 | 0.029 | 0.005 | 9.10E-08 | 0.0012 | 0.0368 | 0.975 |
| rs3172494 | 3 | 4.90E+07 | T | G | 0.1157 | 0.026 | 0.004 | 4.90E-11 | 0.0642 | 0.0253 | 0.01131 |
| rs4076457 | 15 | 7.80E+07 | T | C | 0.2313 | 0.015 | 0.003 | 3.00E-07 | -0.0055 | 0.0185 | 0.7645 |
| rs12076635 | 1 | 4.40E+07 | C | G | 0.7556 | 0.018 | 0.003 | 1.70E-09 | 0.0124 | 0.0192 | 0.5202 |
| rs12646808 | 4 | 3.20E+06 | T | C | 0.6418 | 0.013 | 0.003 | 4.40E-07 | - | - | - |
| rs4851251 | 2 | 1.00E+08 | C | T | 0.2537 | 0.015 | 0.003 | 2.80E-08 | -0.0251 | 0.0182 | 0.1668 |
| rs12531458 | 7 | 3.90E+07 | A | C | 0.5354 | 0.011 | 0.002 | 3.50E-06 | 0.0004 | 0.0155 | 0.9783 |
| rs142328051 | 1 | 4.40E+07 | T | C | 0.8955 | 0.024 | 0.004 | 3.20E-08 | - | - | - |
| rs12143094 | 1 | 7.20E+07 | C | G | 0.03731 | 0.032 | 0.005 | 4.40E-09 | -0.147 | 0.0833 | 0.07745 |
| rs1562242 | 5 | 5.80E+07 | C | T | 0.472 | 0.014 | 0.002 | 7.00E-09 | -0.0079 | 0.0156 | 0.6108 |
| rs4675248 | 2 | 2.00E+08 | G | A | 0.3507 | 0.013 | 0.003 | 6.50E-07 | -0.0058 | 0.0159 | 0.7167 |
| rs7948975 | 11 | 9.00E+07 | T | C | 0.6586 | 0.014 | 0.003 | 3.80E-08 | 0.0349 | 0.0183 | 0.05666 |
| rs1389473 | 12 | 9.20E+07 | G | A | 0.2817 | 0.013 | 0.003 | 1.60E-07 | -0.0136 | 0.0166 | 0.4126 |
| rs9401593 | 6 | 9.90E+07 | C | A | 0.4963 | 0.024 | 0.002 | 1.70E-21 | -0.0231 | 0.0156 | 0.1393 |
| rs320700 | 7 | 1.40E+08 | A | G | 0.6343 | 0.016 | 0.003 | 1.50E-09 | -0.005 | 0.0162 | 0.7554 |
| rs7593947 | 2 | 6.10E+07 | A | T | 0.5243 | 0.015 | 0.002 | 5.20E-10 | -0.0368 | 0.0163 | 0.0236 |
| rs148490894 | 7 | 1.00E+08 | A | G | 0.98507 | 0.048 | 0.009 | 3.20E-08 | - | - | - |
| rs12142680 | 1 | 7.40E+07 | A | G | 0.08396 | 0.027 | 0.005 | 1.40E-08 | -0.0214 | 0.0597 | 0.7203 |
| rs12754946 | 1 | 7.80E+07 | T | C | 0.6119 | 0.012 | 0.002 | 1.80E-06 | -0.0155 | 0.0166 | 0.3505 |
| rs56158183 | 2 | 6.10E+07 | A | G | 0.06343 | 0.022 | 0.005 | 3.10E-06 | -0.0595 | 0.035 | 0.08928 |
| rs11756123 | 6 | 1.50E+08 | T | A | 0.3228 | 0.013 | 0.003 | 2.50E-07 | -0.0395 | 0.0165 | 0.01628 |
| rs76076331 | 2 | 1.10E+07 | T | C | 0.09328 | 0.018 | 0.004 | 3.70E-07 | 0.0054 | 0.024 | 0.823 |
| rs1382358 | 19 | 1.30E+07 | T | C | 0.90672 | 0.021 | 0.004 | 3.20E-08 | -0.0336 | 0.0283 | 0.2353 |
| rs56262138 | 3 | 8.60E+07 | A | T | 0.2668 | 0.012 | 0.003 | 0.000019 | 0.0029 | 0.0194 | 0.8828 |
| rs17504614 | 2 | 5.10E+07 | T | C | 0.8172 | 0.015 | 0.003 | 6.40E-07 | -0.0629 | 0.021 | 0.002775 |
| rs113011189 | 3 | 4.90E+07 | C | T | 0.0653 | 0.027 | 0.005 | 9.00E-08 | -0.0513 | 0.0618 | 0.4063 |
| rs1378214 | 15 | 4.80E+07 | C | T | 0.3825 | 0.016 | 0.003 | 1.20E-10 | -0.0242 | 0.0159 | 0.1288 |
| rs1338554 | 6 | 9.80E+07 | A | G | 0.5299 | 0.016 | 0.002 | 3.60E-10 | -0.0142 | 0.0157 | 0.365 |
| rs71413877 | 2 | 1.00E+08 | A | G | 0.03731 | 0.036 | 0.006 | 1.50E-08 | -0.0035 | 0.0921 | 0.9697 |
| rs9556958 | 13 | 9.90E+07 | C | T | 0.5019 | 0.015 | 0.002 | 1.90E-09 | 0.025 | 0.0158 | 0.1142 |
| rs1115240 | 14 | 2.70E+07 | G | C | 0.7444 | 0.014 | 0.003 | 3.40E-07 | -0.012 | 0.0177 | 0.4998 |
| rs71326918 | 3 | 5.00E+07 | A | C | 0.1045 | 0.026 | 0.004 | 3.20E-09 | 0.0021 | 0.0384 | 0.9562 |
| rs12145291 | 1 | 7.40E+07 | C | T | 0.93657 | 0.027 | 0.006 | 1.60E-06 | -0.0491 | 0.034 | 0.1487 |
| rs12462428 | 19 | 1.70E+07 | T | C | 0.8209 | 0.016 | 0.003 | 2.50E-07 | -0.0002 | 0.0201 | 0.9917 |
| rs61160187 | 5 | 6.00E+07 | G | A | 0.6194 | 0.018 | 0.003 | 5.90E-13 | -0.0267 | 0.0163 | 0.1007 |
| rs62263923 | 3 | 8.60E+07 | G | A | 0.6437 | 0.016 | 0.003 | 1.60E-09 | 0.0057 | 0.0161 | 0.7243 |
| rs2179152 | 6 | 2.60E+07 | C | T | 0.3918 | 0.014 | 0.003 | 1.90E-08 | -0.0213 | 0.0167 | 0.2035 |
| rs7286601 | 22 | 5.10E+07 | G | T | 0.5429 | 0.015 | 0.003 | 3.60E-09 | 0.008 | 0.0188 | 0.6713 |
| rs12410444 | 1 | 4.40E+07 | G | A | 0.7183 | 0.018 | 0.003 | 2.10E-11 | -0.0124 | 0.0171 | 0.4705 |
| rs356992 | 2 | 6.10E+07 | C | G | 0.3284 | 0.017 | 0.003 | 4.20E-10 | -0.0369 | 0.0173 | 0.03341 |
| rs111730030 | 19 | 1.30E+07 | G | T | 0.04664 | 0.030 | 0.006 | 4.80E-08 | - | - | - |
| rs7964899 | 12 | 1.50E+07 | A | G | 0.4571 | 0.017 | 0.002 | 2.00E-11 | -0.0162 | 0.0156 | 0.2967 |
| rs62262721 | 3 | 5.00E+07 | T | C | 0.97948 | 0.035 | 0.009 | 0.00005 | - | - | - |
| rs10772644 | 12 | 1.30E+07 | C | G | 0.8713 | 0.021 | 0.004 | 4.10E-08 | 0.0048 | 0.0255 | 0.8508 |
| rs7633857 | 3 | 1.60E+08 | G | C | 0.5093 | 0.015 | 0.003 | 4.60E-07 | 0.0183 | 0.0156 | 0.2404 |
| rs10773002 | 12 | 1.20E+08 | A | T | 0.2724 | 0.023 | 0.003 | 1.00E-15 | -0.0454 | 0.0177 | 0.01052 |
| rs11771168 | 7 | 1.10E+08 | C | T | 0.2593 | 0.015 | 0.003 | 6.10E-07 | 0.0171 | 0.019 | 0.3685 |
| rs6493271 | 15 | 4.80E+07 | T | C | 0.8414 | 0.017 | 0.003 | 1.10E-07 | 0.0125 | 0.0204 | 0.5397 |
| rs10821136 | 9 | 9.60E+07 | T | C | 0.3246 | 0.012 | 0.003 | 2.40E-06 | -0.0149 | 0.0163 | 0.3607 |
| rs10483349 | 14 | 3.00E+07 | G | A | 0.8302 | 0.019 | 0.003 | 3.00E-09 | -0.0258 | 0.0199 | 0.1953 |
| rs10178115 | 2 | 1.60E+08 | T | G | 0.5205 | 0.014 | 0.002 | 1.00E-08 | -0.007 | 0.0158 | 0.6597 |
| rs111321694 | 11 | 1.10E+08 | C | T | 0.1828 | 0.018 | 0.003 | 4.20E-08 | 0.0036 | 0.0239 | 0.8796 |
| rs11130222 | 3 | 5.00E+07 | A | T | 0.5765 | 0.026 | 0.003 | 4.60E-25 | 0.0129 | 0.0157 | 0.4097 |
| rs4500960 | 2 | 1.60E+08 | C | T | 0.4776 | 0.014 | 0.002 | 6.60E-09 | -0.0233 | 0.016 | 0.1454 |
| rs4741351 | 9 | 1.40E+07 | G | A | 0.3097 | 0.017 | 0.003 | 1.10E-09 | -0.0092 | 0.0182 | 0.6145 |
| rs62100765 | 18 | 5.10E+07 | C | T | 0.403 | 0.014 | 0.002 | 1.10E-08 | 0.0263 | 0.0158 | 0.09635 |
| rs660001 | 5 | 1.10E+08 | G | A | 0.2351 | 0.018 | 0.003 | 1.30E-09 | 0.0022 | 0.0194 | 0.9102 |
| rs10818606 | 9 | 1.20E+08 | C | T | 0.3843 | 0.013 | 0.003 | 1.70E-07 | -0.0085 | 0.0161 | 0.5963 |
| rs17372140 | 1 | 9.90E+07 | G | A | 0.3172 | 0.015 | 0.003 | 6.40E-08 | -0.0162 | 0.0174 | 0.3498 |
| rs149613931 | 10 | 1.00E+08 | G | T | 0.06903 | 0.030 | 0.005 | 1.50E-08 | -0.0538 | 0.0341 | 0.1145 |
| rs7590368 | 2 | 1.10E+07 | C | T | 0.7369 | 0.013 | 0.003 | 5.30E-06 | -0.0007 | 0.0173 | 0.9685 |
| rs17824247 | 2 | 1.40E+08 | C | T | 0.5802 | 0.018 | 0.003 | 5.30E-13 | 0.0031 | 0.0158 | 0.8436 |
| rs1008078 | 1 | 9.10E+07 | C | T | 0.3731 | 0.016 | 0.003 | 7.90E-11 | -0.0364 | 0.0163 | 0.02584 |
| rs12134151 | 1 | 9.60E+07 | G | C | 0.4813 | 0.013 | 0.002 | 1.00E-07 | 0.0099 | 0.0157 | 0.527 |
| rs34344888 | 14 | 2.30E+07 | G | A | 0.3974 | 0.016 | 0.003 | 1.10E-10 | -0.0002 | 0.0159 | 0.9921 |
| rs4308415 | 4 | 6.80E+07 | G | C | 0.4925 | 0.012 | 0.002 | 4.90E-07 | -0.0049 | 0.0155 | 0.7513 |
| rs9755467 | 3 | 1.30E+08 | T | C | 0.1735 | 0.019 | 0.003 | 2.70E-08 | -0.0243 | 0.0205 | 0.2367 |
| rs7241530 | 18 | 7.60E+07 | C | T | 0.3228 | 0.013 | 0.003 | 5.30E-07 | -0.0161 | 0.0192 | 0.4027 |
| rs34106693 | 2 | 1.00E+08 | C | G | 0.819 | 0.016 | 0.003 | 1.70E-06 | -0.0062 | 0.0233 | 0.7897 |
| rs281302 | 15 | 4.80E+07 | G | A | 0.5746 | 0.014 | 0.002 | 6.10E-08 | -0.0108 | 0.0161 | 0.5023 |
| rs35771425 | 1 | 2.10E+08 | T | C | 0.7948 | 0.019 | 0.003 | 2.60E-10 | -0.0007 | 0.0187 | 0.9693 |
| rs756912 | 7 | 7.20E+07 | C | T | 0.5784 | 0.012 | 0.002 | 5.30E-07 | - | - | - |
| rs13090388 | 3 | 4.90E+07 | T | C | 0.2966 | 0.027 | 0.003 | 4.30E-23 | -0.0082 | 0.0174 | 0.6394 |
| rs76878669 | 11 | 6.60E+07 | C | G | 0.7519 | 0.014 | 0.003 | 2.10E-06 | - | - | - |
| rs11774212 | 8 | 1.50E+08 | T | C | 0.4813 | 0.015 | 0.003 | 2.90E-09 | -0.0157 | 0.0174 | 0.3658 |
| rs7945718 | 11 | 1.30E+07 | A | G | 0.5951 | 0.014 | 0.003 | 2.90E-08 | -0.0583 | 0.0169 | 0.000581 |
| rs1912528 | 4 | 1.40E+08 | T | C | 0.3545 | 0.016 | 0.003 | 5.40E-10 | -0.0041 | 0.0161 | 0.8005 |
| rs11998763 | 9 | 1.80E+06 | A | G | 0.556 | 0.017 | 0.002 | 8.40E-12 | -0.0065 | 0.0157 | 0.6792 |
| rs7791133 | 7 | 1.40E+08 | C | A | 0.3657 | 0.013 | 0.003 | 1.40E-07 | 0.0008 | 0.0161 | 0.962 |
| rs1106761 | 8 | 1.40E+08 | G | A | 0.3601 | 0.017 | 0.003 | 4.10E-11 | - | - | - |
| rs78365243 | 1 | 2.10E+08 | T | C | 0.95709 | 0.029 | 0.006 | 3.30E-07 | 0.0214 | 0.0376 | 0.5695 |
| rs1967109 | 4 | 2.90E+07 | G | A | 0.1847 | 0.014 | 0.003 | 0.00007 | -0.0051 | 0.0215 | 0.8125 |
| rs62379838 | 5 | 1.20E+08 | T | C | 0.681 | 0.012 | 0.003 | 4.00E-06 | 0.001 | 0.0172 | 0.9554 |
| rs8002014 | 13 | 5.80E+07 | G | A | 0.2201 | 0.023 | 0.003 | 6.60E-17 | 0.0034 | 0.0185 | 0.8536 |
| rs1550973 | 11 | 1.30E+08 | G | A | 0.3638 | 0.014 | 0.003 | 5.30E-08 | -0.0141 | 0.0171 | 0.4072 |
| rs35532491 | 22 | 3.40E+07 | T | A | 0.8825 | 0.021 | 0.004 | 8.70E-07 | 0.0146 | 0.0258 | 0.5721 |
| rs7429990 | 3 | 4.80E+07 | C | A | 0.2705 | 0.015 | 0.003 | 2.50E-07 | 0.0349 | 0.0176 | 0.04734 |
| rs73344830 | 10 | 1.00E+08 | A | G | 0.4011 | 0.015 | 0.003 | 4.10E-09 | 0.0043 | 0.0161 | 0.7884 |
| rs62263033 | 3 | 4.80E+07 | T | C | 0.96642 | 0.031 | 0.007 | 0.000011 | -0.1114 | 0.0796 | 0.1619 |
| rs12987662 | 2 | 1.00E+08 | A | C | 0.3787 | 0.022 | 0.003 | 3.30E-18 | -0.0238 | 0.0159 | 0.1346 |
| rs648163 | 1 | 2.00E+08 | T | C | 0.2929 | 0.015 | 0.003 | 2.20E-07 | -0.0007 | 0.0178 | 0.9693 |
| rs301800 | 1 | 8.50E+06 | T | C | 0.1791 | 0.017 | 0.003 | 2.40E-07 | -0.0052 | 0.0195 | 0.7891 |
| rs10223052 | 5 | 6.10E+07 | A | G | 0.3657 | 0.018 | 0.003 | 4.60E-12 | -0.0217 | 0.0164 | 0.1856 |
| rs79925071 | 11 | 1.20E+08 | T | G | 0.5168 | 0.012 | 0.002 | 1.30E-06 | 0.0142 | 0.0178 | 0.4256 |
| rs700590 | 5 | 8.80E+07 | C | T | 0.5896 | 0.013 | 0.003 | 5.00E-07 | -0.0175 | 0.0158 | 0.2695 |
| rs1925576 | 10 | 6.90E+07 | G | A | 0.5466 | 0.012 | 0.002 | 2.50E-06 | -0.0347 | 0.0155 | 0.02498 |
| rs12534506 | 7 | 9.30E+07 | T | A | 0.4534 | 0.015 | 0.003 | 7.90E-09 | - | - | - |
| rs6839705 | 4 | 1.10E+08 | A | C | 0.3601 | 0.017 | 0.003 | 1.70E-11 | -0.0015 | 0.0161 | 0.9245 |
| rs1596747 | 2 | 1.90E+08 | A | G | 0.5205 | 0.012 | 0.002 | 4.20E-07 | -0.0289 | 0.0158 | 0.06705 |
| rs6882046 | 5 | 8.80E+07 | G | A | 0.6866 | 0.021 | 0.003 | 7.90E-14 | -0.0436 | 0.017 | 0.0103 |
| rs113474297 | 5 | 6.10E+07 | C | T | 0.1231 | 0.023 | 0.004 | 1.50E-09 | -0.0478 | 0.0231 | 0.03798 |
| rs4741343 | 9 | 1.40E+07 | G | A | 0.1698 | 0.015 | 0.003 | 1.90E-06 | 0.0011 | 0.0206 | 0.9558 |
| rs6939294 | 6 | 1.70E+07 | T | C | 0.2612 | 0.015 | 0.003 | 2.00E-07 | -0.0282 | 0.0177 | 0.1113 |
| rs58694847 | 14 | 8.50E+07 | G | C | 0.3097 | 0.018 | 0.003 | 7.40E-11 | 0.0125 | 0.0177 | 0.4799 |
| rs78387210 | 20 | 4.80E+07 | T | C | 0.09888 | 0.023 | 0.004 | 3.10E-07 | - | - | - |
| rs7605827 | 2 | 1.60E+07 | A | T | 0.2407 | 0.017 | 0.003 | 3.60E-07 | - | - | - |
| rs2568955 | 1 | 7.30E+07 | C | T | 0.2369 | 0.015 | 0.003 | 1.80E-07 | -0.0241 | 0.0193 | 0.2124 |
| rs4378243 | 1 | 9.80E+07 | T | G | 0.8507 | 0.018 | 0.003 | 2.30E-08 | -0.0056 | 0.0219 | 0.8 |
| rs6065080 | 20 | 6.00E+07 | C | T | 0.347 | 0.012 | 0.003 | 2.90E-06 | 0.0116 | 0.0167 | 0.487 |
| rs113520408 | 7 | 1.30E+08 | A | G | 0.2668 | 0.013 | 0.003 | 6.60E-06 | 0.0148 | 0.0175 | 0.3991 |
| rs113779084 | 7 | 1.20E+07 | A | G | 0.2631 | 0.011 | 0.003 | 0.000043 | 0.0036 | 0.0172 | 0.8331 |
| rs12694681 | 2 | 2.30E+08 | T | G | 0.7313 | 0.014 | 0.003 | 9.80E-08 | 0.0335 | 0.0176 | 0.05699 |
| rs12702087 | 7 | 4.50E+07 | A | G | 0.4627 | 0.013 | 0.002 | 2.80E-07 | 0.0109 | 0.0155 | 0.4819 |
| rs55786114 | 3 | 4.90E+07 | C | T | 0.07463 | 0.028 | 0.005 | 3.80E-08 | 0.0393 | 0.0387 | 0.3105 |
| rs7772172 | 6 | 1.70E+07 | A | G | 0.3787 | 0.014 | 0.003 | 5.90E-08 | 0.0099 | 0.0156 | 0.5251 |
| rs6715849 | 2 | 1.00E+08 | G | A | 0.4776 | 0.015 | 0.003 | 1.20E-09 | 0.0175 | 0.0156 | 0.2616 |
| rs11687170 | 2 | 2.40E+08 | T | C | 0.8284 | 0.024 | 0.004 | 3.50E-09 | -0.0321 | 0.0214 | 0.134 |
| rs9914544 | 17 | 1.90E+07 | C | A | 0.6194 | 0.013 | 0.003 | 6.40E-07 | 0.0154 | 0.0159 | 0.3341 |
| rs56231335 | 6 | 9.80E+07 | C | T | 0.6474 | 0.016 | 0.003 | 1.60E-09 | -0.0471 | 0.0187 | 0.01174 |
| rs4493682 | 5 | 4.50E+07 | C | G | 0.2034 | 0.019 | 0.003 | 2.30E-08 | -0.0041 | 0.02 | 0.8389 |
| rs2624818 | 3 | 5.00E+07 | A | G | 0.1063 | 0.021 | 0.004 | 4.50E-07 | -0.057 | 0.0266 | 0.03186 |
| rs9964724 | 18 | 3.50E+07 | T | C | 0.6418 | 0.018 | 0.003 | 1.20E-11 | -0.0023 | 0.0171 | 0.893 |
| rs1606974 | 2 | 5.20E+07 | A | G | 0.1101 | 0.021 | 0.004 | 4.00E-08 | -0.0226 | 0.0259 | 0.384 |
| rs77702819 | 2 | 1.00E+08 | T | G | 0.08582 | 0.022 | 0.004 | 4.40E-07 | 0.0039 | 0.0305 | 0.8973 |
| rs10761741 | 10 | 6.50E+07 | T | G | 0.431 | 0.015 | 0.003 | 5.60E-09 | -0.0221 | 0.0156 | 0.1561 |
| rs192818565 | 17 | 4.40E+07 | T | G | 0.7388 | 0.019 | 0.003 | 7.70E-09 | - | - | - |
| rs61874768 | 10 | 1.00E+08 | G | T | 0.1735 | 0.017 | 0.003 | 1.60E-07 | -0.0049 | 0.0209 | 0.8146 |
| rs11976020 | 7 | 7.20E+07 | G | A | 0.25 | 0.015 | 0.003 | 4.70E-07 | 0.0028 | 0.0195 | 0.8859 |
| rs775326 | 5 | 6.30E+07 | C | A | 0.3116 | 0.013 | 0.003 | 2.20E-06 | - | - | - |
| rs12653396 | 5 | 8.80E+07 | T | A | 0.5168 | 0.013 | 0.002 | 2.10E-07 | 0.0006 | 0.0155 | 0.9716 |
| rs7914680 | 10 | 6.80E+07 | G | T | 0.6791 | 0.015 | 0.003 | 1.10E-07 | 0.0071 | 0.017 | 0.6768 |
| rs112634398 | 3 | 5.00E+07 | A | G | 0.95896 | 0.041 | 0.006 | 2.40E-11 | - | - | - |
| rs7776010 | 6 | 1.50E+07 | C | T | 0.7537 | 0.021 | 0.003 | 3.00E-10 | - | - | - |
| rs35971989 | 3 | 5.10E+07 | A | G | 0.8172 | 0.018 | 0.004 | 6.00E-07 | - | - | - |
| rs12640626 | 4 | 1.80E+08 | A | G | 0.5821 | 0.013 | 0.002 | 8.70E-08 | 0.014 | 0.0158 | 0.374 |
| rs268134 | 2 | 6.60E+07 | A | G | 0.2351 | 0.015 | 0.003 | 1.90E-07 | 0.0482 | 0.0178 | 0.006645 |
| rs75090987 | 5 | 1.00E+08 | A | C | 0.5243 | 0.015 | 0.002 | 5.80E-09 | - | - | - |
| rs56044892 | 1 | 4.20E+07 | C | T | 0.1903 | 0.015 | 0.003 | 1.10E-06 | -0.0076 | 0.023 | 0.7425 |
| rs2406253 | 7 | 1.00E+08 | A | G | 0.8153 | 0.017 | 0.003 | 5.70E-08 | - | - | - |
| rs7610856 | 3 | 7.20E+07 | A | C | 0.4123 | 0.013 | 0.003 | 3.10E-07 | 0.0059 | 0.0157 | 0.7081 |
| rs2456973 | 12 | 5.60E+07 | C | A | 0.6791 | 0.018 | 0.003 | 1.60E-12 | -0.015 | 0.0164 | 0.362 |
| rs2992632 | 1 | 2.40E+08 | A | T | 0.6604 | 0.017 | 0.003 | 7.60E-10 | -0.0468 | 0.0178 | 0.008673 |
| rs17425572 | 9 | 8.80E+07 | A | G | 0.4403 | 0.014 | 0.002 | 4.60E-08 | -0.0039 | 0.0156 | 0.8027 |
| rs11588857 | 1 | 2.00E+08 | A | G | 0.209 | 0.022 | 0.003 | 1.30E-12 | 0.0085 | 0.0186 | 0.648 |
| rs7033137 | 9 | 7.20E+07 | C | G | 0.7743 | 0.016 | 0.003 | 2.10E-08 | 0.0063 | 0.0203 | 0.756 |
| rs10786662 | 10 | 1.00E+08 | G | C | 0.5466 | 0.017 | 0.002 | 3.70E-12 | 0.0157 | 0.0155 | 0.3125 |
| rs34305371 | 1 | 7.30E+07 | A | G | 0.08769 | 0.036 | 0.004 | 2.30E-16 | -0.0531 | 0.0356 | 0.1362 |
| rs8049439 | 16 | 2.90E+07 | T | C | 0.6549 | 0.015 | 0.003 | 2.70E-09 | 0.0196 | 0.0163 | 0.2298 |
| rs12761761 | 10 | 1.30E+08 | T | C | 0.2071 | 0.017 | 0.003 | 3.20E-08 | -0.0104 | 0.0194 | 0.5908 |
| rs12900061 | 15 | 6.60E+07 | A | G | 0.1623 | 0.021 | 0.003 | 2.50E-10 | -0.0069 | 0.0207 | 0.7383 |
| rs28420834 | 15 | 8.30E+07 | G | A | 0.4291 | 0.015 | 0.003 | 1.60E-09 | -0.0296 | 0.0168 | 0.07839 |

SNP – single nucleotide polymorphism; CHR – chromosome’ POS- position; A1 – effect allele 1; A2 – other allele 2; EAF – effect allele frequency; BETA - beta coefficient for association between each SNP and years of schooling; SE – standard error for associations with each SNP; P – P value for associations with each SNP; LOR – Log odds ratios for the association between each SNP Alzheimer’s disease

**Table S2: List of SNPs used from the intelligence GWAS**

|  |  |  |  |  |  | **Associations with intelligence** | | | **Associations with Alzheimer’s disease** | | |
| --- | --- | --- | --- | --- | --- | --- | --- | --- | --- | --- | --- |
| **SNP** | **CHR** | **POS** | **A1** | **A2** | **EAF** | **BETA** | **SE** | **P** | **LOR** | **SE** | **P** |
| rs11664298 | 18 | 7.80E+07 | G | A | 0.794 | 0.023153 | 0.0037 | 3.80E-10 | 0.033 | 0.0208 | 0.1116 |
| rs13220261 | 6 | 2.60E+07 | T | C | 0.8852 | 0.0363201 | 0.0047 | 9.70E-15 | -0.0254 | 0.0302 | 0.401 |
| rs1363119 | 19 | 1.80E+07 | G | A | 0.4503 | 0.0181893 | 0.0031 | 3.60E-09 | -0.0271 | 0.0164 | 0.09929 |
| rs132570 | 22 | 4.00E+07 | A | C | 0.4682 | 0.0201018 | 0.003 | 2.00E-11 | -0.0068 | 0.0162 | 0.6721 |
| rs6979354 | 7 | 2.10E+07 | T | C | 0.4545 | 0.0195457 | 0.0029 | 2.50E-11 | -0.0229 | 0.0161 | 0.1539 |
| rs1972863 | 4 | 9.50E+07 | G | A | 0.6855 | 0.0181282 | 0.0033 | 4.10E-08 | 0.0081 | 0.0167 | 0.6291 |
| rs11022505 | 11 | 1.30E+07 | G | A | 0.4491 | 0.0182448 | 0.0029 | 4.90E-10 | -0.0433 | 0.0156 | 0.005502 |
| rs2944839 | 7 | 7.20E+07 | C | T | 0.4776 | 0.0216613 | 0.0029 | 1.20E-13 | -0.0158 | 0.0156 | 0.3115 |
| rs11793831 | 9 | 2.30E+07 | T | G | 0.5897 | 0.0306136 | 0.0031 | 9.30E-23 | -0.0239 | 0.0173 | 0.1684 |
| rs9906944 | 17 | 4.70E+07 | T | C | 0.6612 | 0.0182853 | 0.0032 | 7.20E-09 | -0.0193 | 0.0167 | 0.2473 |
| rs10511071 | 3 | 8.60E+07 | C | T | 0.8911 | 0.0267859 | 0.0047 | 1.00E-08 | -0.0396 | 0.0266 | 0.1364 |
| rs1376303 | 7 | 3.30E+07 | T | C | 0.1702 | 0.0219726 | 0.0039 | 1.50E-08 | -0.0308 | 0.0221 | 0.1637 |
| rs3735478 | 7 | 4.50E+07 | T | G | 0.7179 | 0.0207866 | 0.0033 | 4.00E-10 | -0.0077 | 0.0192 | 0.6892 |
| rs12693854 | 2 | 2.00E+08 | A | G | 0.5764 | 0.0181256 | 0.003 | 8.10E-10 | 0.0014 | 0.0156 | 0.9287 |
| rs12707116 | 7 | 1.30E+08 | C | T | 0.5185 | 0.0240882 | 0.0029 | 1.50E-16 | -0.0108 | 0.0154 | 0.4808 |
| rs6840360 | 4 | 1.50E+08 | G | A | 0.4995 | 0.0205007 | 0.003 | 7.20E-12 | 0.0279 | 0.0161 | 0.08295 |
| rs1947988 | 10 | 1.10E+08 | C | T | 0.7634 | 0.019306 | 0.0034 | 1.80E-08 | -0.0301 | 0.0182 | 0.09763 |
| rs17426174 | 17 | 4.40E+07 | G | C | 0.7833 | 0.0231488 | 0.0035 | 6.10E-11 | - | - | - |
| rs1481045 | 8 | 7.10E+07 | C | T | 0.827 | 0.0237473 | 0.0039 | 7.30E-10 | -0.0111 | 0.0214 | 0.6027 |
| rs12427622 | 13 | 5.70E+07 | T | C | 0.7763 | 0.0202962 | 0.0037 | 3.50E-08 | -0.0007 | 0.0185 | 0.9683 |
| rs12313068 | 12 | 1.10E+08 | T | C | 0.8403 | 0.0237801 | 0.004 | 2.30E-09 | -0.0047 | 0.0211 | 0.8228 |
| rs17589603 | 1 | 7.30E+07 | A | G | 0.9031 | 0.0288686 | 0.0051 | 1.10E-08 | -0.0448 | 0.0322 | 0.164 |
| rs12552 | 13 | 5.40E+07 | A | G | 0.4332 | 0.0162742 | 0.0029 | 3.20E-08 | -0.0274 | 0.0155 | 0.07772 |
| rs166820 | 5 | 8.90E+07 | A | G | 0.8409 | 0.0224141 | 0.004 | 1.90E-08 | -0.008 | 0.0207 | 0.6985 |
| rs16822665 | 2 | 1.60E+08 | T | C | 0.6951 | 0.0222653 | 0.0032 | 7.20E-12 | 0.0035 | 0.0177 | 0.8451 |
| rs337939 | 13 | 7.10E+07 | T | C | 0.5754 | 0.0163143 | 0.003 | 3.20E-08 | -0.0058 | 0.0158 | 0.7114 |
| rs17033621 | 2 | 1.10E+08 | T | C | 0.5382 | 0.0179264 | 0.0029 | 8.90E-10 | -0.0281 | 0.0155 | 0.06902 |
| rs2887218 | 2 | 1.80E+08 | T | C | 0.5388 | 0.0163887 | 0.0029 | 2.10E-08 | -0.0117 | 0.0155 | 0.45 |
| rs2486012 | 1 | 4.40E+07 | A | G | 0.9032 | 0.0374917 | 0.0051 | 1.20E-13 | -0.0193 | 0.0259 | 0.456 |
| rs17489649 | 5 | 1.10E+08 | A | G | 0.673 | 0.018599 | 0.0031 | 2.20E-09 | -0.0213 | 0.0163 | 0.1911 |
| rs4132462 | 2 | 2.10E+08 | C | T | 0.6991 | 0.018753 | 0.0033 | 8.80E-09 | -0.025 | 0.0172 | 0.1476 |
| rs1389993 | 21 | 1.90E+07 | C | G | 0.5895 | 0.0189414 | 0.0031 | 1.20E-09 | -0.0011 | 0.0163 | 0.9443 |
| rs1045902 | 2 | 7.30E+07 | T | C | 0.5122 | 0.0195449 | 0.003 | 6.50E-11 | -0.0169 | 0.0171 | 0.3248 |
| rs1650007 | 12 | 4.60E+07 | T | C | 0.4792 | 0.0173171 | 0.0031 | 1.70E-08 | 0.0108 | 0.0174 | 0.5324 |
| rs1360123 | 10 | 2.60E+07 | G | A | 0.5148 | 0.0176334 | 0.0031 | 9.10E-09 | -0.0197 | 0.0154 | 0.1991 |
| rs1297620 | 7 | 1.00E+08 | G | A | 0.109 | 0.0292304 | 0.0049 | 2.80E-09 | -0.0683 | 0.025 | 0.006223 |
| rs11579557 | 1 | 2.10E+08 | G | A | 0.7817 | 0.0214852 | 0.0035 | 1.20E-09 | -0.001 | 0.0189 | 0.9568 |
| rs17049820 | 2 | 5.90E+07 | C | T | 0.8839 | 0.0255095 | 0.0047 | 4.70E-08 | -0.0322 | 0.0264 | 0.223 |
| rs78382112 | 1 | 1.80E+08 | A | G | 0.9504 | 0.0449681 | 0.0071 | 1.90E-10 | 0.0375 | 0.0371 | 0.3132 |
| rs6731420 | 2 | 7.20E+07 | C | T | 0.4358 | 0.0164387 | 0.0029 | 2.30E-08 | -0.0377 | 0.0157 | 0.01657 |
| rs1479119 | 12 | 1.40E+07 | G | A | 0.5687 | 0.0179205 | 0.0029 | 1.10E-09 | -0.0066 | 0.0156 | 0.6708 |
| rs12519613 | 5 | 6.80E+07 | C | T | 0.8449 | 0.0231701 | 0.0041 | 2.00E-08 | 0.0125 | 0.0263 | 0.6332 |
| rs72828517 | 6 | 1.90E+07 | C | T | 0.8244 | 0.0223962 | 0.004 | 2.80E-08 | -0.0055 | 0.0204 | 0.7872 |
| rs10189857 | 2 | 6.10E+07 | A | G | 0.564 | 0.0237934 | 0.0029 | 5.90E-16 | -0.0428 | 0.0163 | 0.008865 |
| rs9862291 | 3 | 1.40E+08 | G | A | 0.5334 | 0.0190869 | 0.0031 | 5.30E-10 | 0.0193 | 0.0154 | 0.2093 |
| rs72904190 | 2 | 1.60E+08 | A | G | 0.892 | 0.0284595 | 0.0049 | 8.40E-09 | 0.0591 | 0.026 | 0.0229 |
| rs10236197 | 7 | 3.20E+07 | T | C | 0.6253 | 0.0184345 | 0.003 | 9.40E-10 | -0.0255 | 0.0164 | 0.1193 |
| rs12477602 | 2 | 2.00E+08 | G | A | 0.8671 | 0.0244115 | 0.0043 | 1.90E-08 | - | - | - |
| rs11083241 | 18 | 2.60E+07 | G | A | 0.402 | 0.0170368 | 0.003 | 1.00E-08 | -0.0101 | 0.0156 | 0.5177 |
| rs1318878 | 12 | 1.60E+07 | C | A | 0.7922 | 0.0216687 | 0.0038 | 9.80E-09 | -0.0052 | 0.019 | 0.7822 |
| rs5770820 | 22 | 5.10E+07 | G | A | 0.7712 | 0.0204655 | 0.0035 | 3.70E-09 | 0.0211 | 0.0204 | 0.3006 |
| rs2292989 | 1 | 3.20E+07 | A | G | 0.6445 | 0.0234297 | 0.003 | 1.50E-14 | -0.0186 | 0.0163 | 0.2542 |
| rs72759215 | 5 | 6.00E+07 | T | C | 0.5621 | 0.021699 | 0.0031 | 2.20E-12 | -0.0266 | 0.0156 | 0.08919 |
| rs12206087 | 6 | 9.90E+07 | A | G | 0.5281 | 0.0380856 | 0.0029 | 7.40E-39 | -0.0259 | 0.0156 | 0.09688 |
| rs11665242 | 18 | 5.10E+07 | A | G | 0.5914 | 0.0238597 | 0.003 | 8.70E-16 | 0.0131 | 0.0156 | 0.3997 |
| rs1106241 | 12 | 1.20E+08 | T | C | 0.2231 | 0.0259369 | 0.0035 | 1.30E-13 | -0.0477 | 0.0192 | 0.013 |
| rs12189452 | 5 | 1.10E+08 | G | A | 0.8263 | 0.0224845 | 0.0038 | 5.20E-09 | -0.0164 | 0.0204 | 0.4236 |
| rs7260359 | 19 | 4.60E+07 | C | T | 0.5124 | 0.0167068 | 0.003 | 2.40E-08 | 0.0214 | 0.0169 | 0.2063 |
| rs10498730 | 6 | 2.60E+07 | A | G | 0.9447 | 0.0351255 | 0.0064 | 3.70E-08 | -0.0464 | 0.0345 | 0.179 |
| rs10922907 | 1 | 9.10E+07 | T | A | 0.4465 | 0.0216741 | 0.0031 | 2.10E-12 | -0.0272 | 0.0162 | 0.09205 |
| rs6034019 | 20 | 1.50E+07 | G | A | 0.3167 | 0.0175952 | 0.0032 | 4.40E-08 | 0.0046 | 0.0173 | 0.7908 |
| rs7618501 | 3 | 5.00E+07 | G | A | 0.496 | 0.0359245 | 0.0031 | 1.10E-31 | -0.0009 | 0.0155 | 0.9529 |
| rs2920939 | 8 | 9.30E+07 | A | G | 0.3877 | 0.0172291 | 0.0031 | 2.00E-08 | -0.0065 | 0.0185 | 0.7257 |
| rs13010010 | 2 | 1.00E+08 | T | C | 0.6306 | 0.0276335 | 0.003 | 5.90E-20 | -0.0243 | 0.0165 | 0.1415 |
| rs2026037 | 9 | 2.40E+07 | T | C | 0.8346 | 0.0230477 | 0.0039 | 4.30E-09 | -0.0234 | 0.0222 | 0.2923 |
| rs72843166 | 17 | 6.10E+07 | T | G | 0.8206 | 0.0225883 | 0.004 | 1.60E-08 | -0.0014 | 0.0195 | 0.9432 |
| rs10876864 | 12 | 5.60E+07 | G | A | 0.428 | 0.0207699 | 0.0029 | 1.80E-12 | -0.0022 | 0.0156 | 0.8895 |
| rs3092073 | 20 | 4.50E+07 | G | A | 0.5469 | 0.0169841 | 0.0031 | 2.80E-08 | -0.01 | 0.0156 | 0.5213 |
| rs4984541 | 15 | 9.70E+07 | G | A | 0.7754 | 0.0209756 | 0.0036 | 6.90E-09 | -0.0081 | 0.0209 | 0.6984 |
| rs117893056 | 8 | 8.50E+07 | C | T | 0.8239 | 0.0221963 | 0.004 | 3.50E-08 | 0.0007 | 0.0232 | 0.9768 |
| rs10175405 | 2 | 1.00E+08 | T | G | 0.5925 | 0.019109 | 0.003 | 1.20E-10 | -0.0264 | 0.0165 | 0.1112 |
| rs12993989 | 2 | 1.60E+08 | T | C | 0.4791 | 0.0174868 | 0.0029 | 2.10E-09 | -0.0219 | 0.0154 | 0.1555 |
| rs2721173 | 8 | 1.50E+08 | C | T | 0.5295 | 0.0226038 | 0.0029 | 1.00E-14 | -0.0134 | 0.0167 | 0.4227 |
| rs10192369 | 2 | 1.60E+08 | G | A | 0.5161 | 0.0172105 | 0.0029 | 3.70E-09 | -0.0013 | 0.0157 | 0.9338 |
| rs1710990 | 14 | 7.00E+07 | G | A | 0.2818 | 0.018935 | 0.0032 | 5.20E-09 | 0.0052 | 0.0169 | 0.7575 |
| rs4904523 | 14 | 9.00E+07 | G | A | 0.4897 | 0.019719 | 0.0029 | 1.40E-11 | -0.0072 | 0.0153 | 0.639 |
| rs74944275 | 5 | 1.00E+08 | T | C | 0.9567 | 0.0496984 | 0.0075 | 4.20E-11 | 0.0306 | 0.0469 | 0.5147 |
| rs1998358 | 14 | 3.00E+07 | A | G | 0.1445 | 0.0259848 | 0.0041 | 3.70E-10 | -0.022 | 0.022 | 0.3176 |
| rs6477493 | 9 | 9.90E+07 | T | C | 0.8102 | 0.0228166 | 0.0038 | 2.20E-09 | -0.0062 | 0.0198 | 0.7539 |
| rs10992756 | 9 | 9.60E+07 | C | T | 0.6646 | 0.0197268 | 0.0031 | 1.70E-10 | -0.0182 | 0.0162 | 0.2616 |
| rs2239647 | 14 | 3.30E+07 | C | A | 0.4399 | 0.020585 | 0.0029 | 2.40E-12 | -0.0008 | 0.016 | 0.9604 |
| rs34172651 | 16 | 2.50E+07 | C | T | 0.6894 | 0.0205231 | 0.0033 | 5.90E-10 | 0.013 | 0.0173 | 0.4508 |
| rs7159131 | 14 | 3.70E+07 | G | A | 0.6005 | 0.0173375 | 0.0031 | 3.10E-08 | -0.0137 | 0.0156 | 0.378 |
| rs17043393 | 1 | 2.20E+08 | A | G | 0.833 | 0.0229025 | 0.004 | 1.10E-08 | -0.0076 | 0.0223 | 0.7344 |
| rs1043595 | 7 | 1.30E+08 | A | G | 0.7277 | 0.0206071 | 0.0033 | 3.20E-10 | 0.0145 | 0.0175 | 0.4051 |
| rs9436866 | 1 | 6.90E+07 | C | A | 0.9033 | 0.0307298 | 0.0049 | 4.70E-10 | -0.0237 | 0.0258 | 0.3586 |
| rs2289328 | 15 | 4.10E+07 | G | A | 0.839 | 0.0276511 | 0.004 | 3.20E-12 | -0.0123 | 0.0209 | 0.5564 |
| rs12155874 | 8 | 1.40E+07 | G | A | 0.5054 | 0.0175471 | 0.003 | 4.50E-09 | -0.0212 | 0.016 | 0.1851 |
| rs11748023 | 5 | 1.40E+08 | T | C | 0.4565 | 0.0201734 | 0.0031 | 5.70E-11 | -0.0681 | 0.0154 | 0.00001 |
| rs78715178 | 3 | 1.10E+08 | A | T | 0.9527 | 0.0418154 | 0.0072 | 7.10E-09 | 0.0547 | 0.0389 | 0.1604 |
| rs17123764 | 12 | 5.00E+07 | T | C | 0.925 | 0.0320926 | 0.0055 | 6.70E-09 | -0.0135 | 0.0321 | 0.6742 |
| rs10191758 | 2 | 1.40E+08 | G | A | 0.619 | 0.0228341 | 0.0032 | 4.80E-13 | 0.0112 | 0.0159 | 0.4809 |
| rs72712511 | 4 | 1.40E+08 | C | A | 0.6785 | 0.0199134 | 0.0033 | 1.30E-09 | 0.0108 | 0.0176 | 0.5399 |
| rs10874938 | 1 | 9.60E+07 | A | T | 0.5276 | 0.0257168 | 0.003 | 9.10E-18 | 0.0053 | 0.0156 | 0.7334 |
| rs2743461 | 22 | 4.30E+07 | A | G | 0.5392 | 0.0184331 | 0.0031 | 2.10E-09 | -0.0207 | 0.0166 | 0.2141 |
| rs11703032 | 22 | 3.40E+07 | T | C | 0.9018 | 0.0280633 | 0.0049 | 1.00E-08 | 0.0148 | 0.0257 | 0.5644 |
| rs971681 | 14 | 3.00E+07 | C | T | 0.6083 | 0.0176184 | 0.003 | 3.70E-09 | -0.0395 | 0.0159 | 0.01298 |
| rs13089785 | 3 | 1.20E+08 | C | A | 0.3766 | 0.0172769 | 0.0031 | 2.20E-08 | -0.0256 | 0.016 | 0.1098 |
| rs2591576 | 5 | 1.70E+08 | T | C | 0.5461 | 0.0185367 | 0.0029 | 2.50E-10 | -0.0186 | 0.0157 | 0.2356 |
| rs34811474 | 4 | 2.50E+07 | A | G | 0.7777 | 0.0234388 | 0.0037 | 2.10E-10 | - | - | - |
| rs7621362 | 3 | 1.30E+08 | C | T | 0.8381 | 0.0231193 | 0.004 | 5.20E-09 | -0.0264 | 0.0217 | 0.225 |
| rs12026245 | 1 | 1.00E+08 | G | A | 0.4933 | 0.0161036 | 0.0029 | 3.40E-08 | -0.0267 | 0.0157 | 0.08766 |
| rs7015263 | 8 | 8.80E+07 | C | A | 0.4868 | 0.0171792 | 0.0031 | 2.20E-08 | -0.0134 | 0.0157 | 0.3935 |
| rs254781 | 5 | 8.80E+07 | C | T | 0.4271 | 0.0293461 | 0.0031 | 2.90E-21 | -0.0342 | 0.0156 | 0.02894 |
| rs12600121 | 16 | 7.20E+07 | G | C | 0.4728 | 0.0183572 | 0.003 | 5.00E-10 | -0.0034 | 0.0154 | 0.823 |
| rs1918202 | 12 | 8.00E+07 | G | A | 0.5837 | 0.0208857 | 0.0031 | 1.90E-11 | 0.01 | 0.0164 | 0.5411 |
| rs9527727 | 13 | 5.80E+07 | C | A | 0.2988 | 0.0233392 | 0.0034 | 3.30E-12 | 0.0079 | 0.0172 | 0.6472 |
| rs11142387 | 9 | 7.30E+07 | A | C | 0.4559 | 0.0163001 | 0.0029 | 2.60E-08 | -0.0146 | 0.0158 | 0.3572 |
| rs11555274 | 19 | 1.30E+07 | G | C | 0.8888 | 0.0273207 | 0.0049 | 2.10E-08 | -0.0416 | 0.0321 | 0.1953 |
| rs2145265 | 20 | 5.80E+07 | T | C | 0.8356 | 0.0254492 | 0.0041 | 7.70E-10 | -0.016 | 0.0219 | 0.4638 |
| rs13262595 | 8 | 1.40E+08 | G | A | 0.4267 | 0.0179888 | 0.003 | 2.70E-09 | -0.0205 | 0.0158 | 0.1942 |
| rs78164635 | 1 | 9.80E+07 | C | T | 0.9238 | 0.0328961 | 0.0058 | 1.30E-08 | -0.0066 | 0.0324 | 0.8389 |
| rs10949662 | 7 | 1.60E+08 | C | T | 0.6553 | 0.0176044 | 0.0031 | 2.20E-08 | 0.0291 | 0.0192 | 0.1289 |
| rs10761739 | 10 | 6.50E+07 | C | G | 0.5835 | 0.0233158 | 0.003 | 3.20E-15 | -0.0221 | 0.0157 | 0.1586 |
| rs9384679 | 6 | 1.10E+08 | C | T | 0.6205 | 0.0271085 | 0.003 | 1.90E-19 | 0.0437 | 0.0165 | 0.008287 |
| rs1054442 | 12 | 4.90E+07 | C | A | 0.6177 | 0.0242621 | 0.0031 | 3.20E-15 | -0.0146 | 0.0173 | 0.4005 |
| rs12035012 | 1 | 4.20E+07 | C | A | 0.7826 | 0.0318854 | 0.0036 | 1.50E-18 | -0.0127 | 0.0191 | 0.5056 |
| rs8025619 | 15 | 5.20E+07 | T | C | 0.9681 | 0.0476602 | 0.0083 | 9.50E-09 | 0.084 | 0.049 | 0.0865 |
| rs6723226 | 2 | 3.30E+07 | A | G | 0.6481 | 0.017146 | 0.0031 | 4.40E-08 | 0.006 | 0.0168 | 0.7199 |
| rs4725065 | 7 | 8.10E+06 | G | A | 0.492 | 0.0184659 | 0.003 | 1.00E-09 | -0.0274 | 0.0154 | 0.07617 |
| rs9655780 | 7 | 1.00E+08 | G | A | 0.1626 | 0.023371 | 0.004 | 3.30E-09 | -0.0243 | 0.0209 | 0.2461 |
| rs12072199 | 1 | 2.10E+07 | C | T | 0.8067 | 0.0234374 | 0.0037 | 2.20E-10 | 0.0018 | 0.0209 | 0.9307 |
| rs11077207 | 16 | 7.70E+06 | G | C | 0.4947 | 0.0184274 | 0.0029 | 2.60E-10 | -0.0335 | 0.0156 | 0.03223 |
| rs9443645 | 6 | 8.00E+07 | C | T | 0.4929 | 0.0180575 | 0.0029 | 6.00E-10 | -0.0241 | 0.016 | 0.1317 |
| rs7309 | 2 | 1.60E+08 | G | A | 0.5094 | 0.0246505 | 0.0029 | 2.90E-17 | 0.0216 | 0.0155 | 0.1639 |
| rs9951698 | 18 | 1.30E+07 | C | G | 0.4332 | 0.017265 | 0.0031 | 2.40E-08 | -0.0267 | 0.0163 | 0.1019 |
| rs2426132 | 20 | 4.80E+07 | C | G | 0.5417 | 0.0277563 | 0.0031 | 1.90E-19 | -0.0073 | 0.0154 | 0.6365 |
| rs862687 | 3 | 7.20E+07 | A | G | 0.5945 | 0.0215856 | 0.0031 | 4.80E-12 | 0.005 | 0.0157 | 0.7527 |
| rs11945232 | 4 | 8.80E+07 | G | A | 0.623 | 0.0169432 | 0.003 | 1.80E-08 | -0.0153 | 0.0158 | 0.3331 |
| rs9388490 | 6 | 1.30E+08 | T | C | 0.5565 | 0.0198815 | 0.003 | 4.00E-11 | -0.0157 | 0.0154 | 0.3081 |
| rs10149470 | 14 | 1.00E+08 | G | A | 0.4873 | 0.0196753 | 0.003 | 4.80E-11 | -0.0198 | 0.0157 | 0.2074 |
| rs6668586 | 1 | 6.00E+07 | A | G | 0.5116 | 0.0179811 | 0.0031 | 4.60E-09 | -0.009 | 0.0157 | 0.5675 |
| rs2731664 | 5 | 1.80E+08 | C | A | 0.4793 | 0.0199179 | 0.003 | 2.90E-11 | 0.009 | 0.0156 | 0.5649 |
| rs72829841 | 6 | 1.70E+07 | G | T | 0.7686 | 0.0216308 | 0.0036 | 2.70E-09 | -0.0268 | 0.0178 | 0.1325 |
| rs620729 | 6 | 1.20E+07 | C | A | 0.6731 | 0.0189176 | 0.0031 | 1.20E-09 | -0.0254 | 0.0163 | 0.118 |
| rs1891275 | 10 | 9.30E+07 | C | T | 0.447 | 0.0181925 | 0.0029 | 5.50E-10 | -0.0046 | 0.0166 | 0.7818 |
| rs17443541 | 2 | 2.00E+08 | T | C | 0.8144 | 0.0218865 | 0.0039 | 2.90E-08 | -0.0288 | 0.0218 | 0.1865 |
| rs10858096 | 1 | 1.10E+08 | C | A | 0.4709 | 0.0212039 | 0.003 | 1.50E-12 | 0.0193 | 0.0155 | 0.2147 |
| rs79839061 | 4 | 705884 | A | G | 0.9233 | 0.0328761 | 0.0058 | 1.20E-08 | 0.0238 | 0.0298 | 0.4254 |
| rs1812587 | 5 | 6.30E+07 | G | T | 0.5423 | 0.0194911 | 0.0031 | 2.40E-10 | 0.0219 | 0.0159 | 0.1691 |
| rs4434138 | 3 | 5.30E+07 | G | A | 0.5512 | 0.0193173 | 0.0031 | 3.70E-10 | -0.0003 | 0.0157 | 0.9834 |
| rs10845988 | 12 | 1.50E+07 | C | T | 0.5692 | 0.0186096 | 0.0029 | 2.60E-10 | -0.0186 | 0.0156 | 0.2311 |
| rs7431278 | 3 | 2.40E+07 | T | C | 0.675 | 0.024832 | 0.0031 | 1.50E-15 | -0.0337 | 0.0169 | 0.04579 |
| rs10897167 | 11 | 6.10E+07 | A | G | 0.9498 | 0.0374096 | 0.0069 | 5.00E-08 | -0.0013 | 0.0448 | 0.9765 |
| rs1421334 | 8 | 3.10E+07 | C | A | 0.458 | 0.018178 | 0.003 | 1.40E-09 | -0.0084 | 0.0161 | 0.6032 |
| rs10211032 | 2 | 1.20E+08 | A | G | 0.4973 | 0.0169581 | 0.0031 | 3.20E-08 | -0.0184 | 0.0155 | 0.2338 |
| rs3935685 | 15 | 7.80E+07 | C | T | 0.5594 | 0.0160794 | 0.0029 | 4.40E-08 | -0.0193 | 0.016 | 0.2282 |
| rs13053308 | 22 | 3.60E+07 | A | G | 0.5387 | 0.0182807 | 0.0031 | 2.80E-09 | 0.0093 | 0.0161 | 0.5638 |
| rs418939 | 5 | 1.10E+08 | T | C | 0.7068 | 0.0176041 | 0.0032 | 3.90E-08 | 0.0008 | 0.0168 | 0.9631 |
| rs6991838 | 8 | 6.60E+07 | A | G | 0.4358 | 0.0173004 | 0.003 | 9.70E-09 | -0.0093 | 0.0159 | 0.5594 |
| rs61774752 | 1 | 4.20E+07 | C | T | 0.4769 | 0.0196384 | 0.0031 | 1.60E-10 | -0.0136 | 0.0161 | 0.4008 |
| rs2155076 | 11 | 9.00E+07 | C | T | 0.7392 | 0.0207167 | 0.0033 | 4.40E-10 | 0.021 | 0.0181 | 0.2459 |
| rs9503598 | 6 | 3.40E+06 | A | G | 0.5567 | 0.0207617 | 0.0031 | 1.30E-11 | 0.0048 | 0.0158 | 0.7631 |
| rs644799 | 11 | 9.60E+07 | G | A | 0.6271 | 0.0184985 | 0.003 | 1.30E-09 | -0.0275 | 0.016 | 0.08575 |
| rs4130344 | 4 | 1.60E+08 | G | T | 0.4294 | 0.0177046 | 0.0029 | 1.90E-09 | -0.0105 | 0.0155 | 0.4965 |
| rs2265097 | 13 | 1.10E+08 | T | G | 0.2557 | 0.0233769 | 0.0033 | 2.70E-12 | -0.0127 | 0.0177 | 0.4713 |
| rs4407350 | 22 | 4.50E+07 | G | A | 0.5286 | 0.016677 | 0.0029 | 1.10E-08 | -0.009 | 0.0155 | 0.5626 |
| rs3744593 | 17 | 3.50E+07 | T | C | 0.5603 | 0.018418 | 0.0029 | 3.60E-10 | -0.0054 | 0.0155 | 0.7296 |
| rs13411363 | 2 | 1.70E+08 | A | G | 0.6433 | 0.0170402 | 0.003 | 2.20E-08 | -0.0313 | 0.0164 | 0.05591 |
| rs2295499 | 4 | 2.70E+06 | C | T | 0.557 | 0.0180981 | 0.0029 | 7.00E-10 | -0.039 | 0.0162 | 0.01611 |
| rs6719830 | 2 | 2.40E+07 | C | T | 0.5488 | 0.0173169 | 0.0029 | 3.40E-09 | -0.0254 | 0.0154 | 0.09888 |
| rs1424638 | 2 | 5.70E+07 | T | C | 0.4605 | 0.0196449 | 0.0029 | 1.90E-11 | -0.0157 | 0.0161 | 0.3317 |
| rs4237845 | 12 | 5.80E+07 | C | T | 0.5379 | 0.0182313 | 0.003 | 1.20E-09 | 0.0091 | 0.0167 | 0.5839 |
| rs67258057 | 6 | 2.20E+07 | G | A | 0.931 | 0.0344557 | 0.006 | 1.20E-08 | -0.0409 | 0.0369 | 0.2682 |
| rs1346081 | 4 | 6.80E+07 | A | G | 0.4427 | 0.0207044 | 0.0029 | 1.80E-12 | 0.0115 | 0.0155 | 0.4571 |
| rs748832 | 3 | 1.70E+07 | A | G | 0.6358 | 0.0192652 | 0.003 | 2.10E-10 | 0.0046 | 0.016 | 0.774 |
| rs12465425 | 2 | 6.80E+07 | G | T | 0.6876 | 0.0190384 | 0.0031 | 1.40E-09 | -0.025 | 0.0172 | 0.1456 |
| rs7157510 | 14 | 6.30E+07 | T | C | 0.3761 | 0.0172883 | 0.0032 | 4.70E-08 | 0.0097 | 0.0159 | 0.5426 |
| rs952623 | 7 | 3.90E+07 | G | C | 0.678 | 0.0182079 | 0.0032 | 1.30E-08 | 0.016 | 0.0208 | 0.4436 |
| rs1566085 | 8 | 1.40E+08 | T | G | 0.458 | 0.0209914 | 0.0031 | 9.10E-12 | 0.0308 | 0.0159 | 0.05292 |
| rs10145335 | 14 | 9.90E+07 | G | A | 0.755 | 0.0206345 | 0.0034 | 1.10E-09 | 0.0027 | 0.0181 | 0.8807 |
| rs9911578 | 17 | 5.70E+07 | T | C | 0.376 | 0.0188875 | 0.003 | 3.50E-10 | -0.0413 | 0.0165 | 0.01211 |
| rs4699954 | 5 | 6.00E+07 | G | A | 0.8189 | 0.0211228 | 0.0038 | 2.40E-08 | -0.0348 | 0.0217 | 0.108 |
| rs10413329 | 19 | 1.30E+07 | A | G | 0.464 | 0.0179263 | 0.003 | 2.30E-09 | -0.0055 | 0.0164 | 0.7369 |
| rs1813006 | 4 | 1.00E+08 | G | T | 0.9421 | 0.0474077 | 0.0064 | 1.50E-13 | - | - | - |
| rs2101975 | 4 | 1.10E+08 | A | G | 0.5598 | 0.0269549 | 0.003 | 3.70E-19 | -0.0281 | 0.0157 | 0.07375 |
| rs10282411 | 7 | 1.30E+08 | C | T | 0.4445 | 0.0210241 | 0.0031 | 9.60E-12 | 0.0129 | 0.0161 | 0.4226 |
| rs13425585 | 2 | 1.60E+08 | G | C | 0.5067 | 0.0219672 | 0.0031 | 8.00E-13 | 0 | 0.0159 | 0.9976 |
| rs350251 | 16 | 1.20E+07 | A | G | 0.4134 | 0.0173884 | 0.0031 | 2.40E-08 | -0.0206 | 0.0158 | 0.1921 |
| rs7189233 | 16 | 5.30E+07 | T | C | 0.6894 | 0.0250169 | 0.0033 | 4.40E-14 | 0.0022 | 0.0165 | 0.8922 |
| rs17425572 | 9 | 8.80E+07 | A | G | 0.4403 | 0.0184279 | 0.0029 | 2.80E-10 | -0.0039 | 0.0156 | 0.8027 |
| rs11588857 | 1 | 2.00E+08 | A | G | 0.209 | 0.023016 | 0.0036 | 1.50E-10 | 0.0085 | 0.0186 | 0.648 |
| rs7033137 | 9 | 7.20E+07 | C | G | 0.7743 | 0.0321583 | 0.0051 | 2.00E-10 | 0.0063 | 0.0203 | 0.756 |
| rs10786662 | 10 | 1.00E+08 | G | C | 0.5466 | 0.0275317 | 0.0031 | 6.40E-19 | 0.0157 | 0.0155 | 0.3125 |
| rs34305371 | 1 | 7.30E+07 | A | G | 0.08769 | 0.0474075 | 0.0052 | 3.90E-20 | -0.0531 | 0.0356 | 0.1362 |
| rs8049439 | 16 | 2.90E+07 | T | C | 0.6549 | 0.0269697 | 0.003 | 1.30E-19 | 0.0196 | 0.0163 | 0.2298 |
| rs12761761 | 10 | 1.30E+08 | T | C | 0.2071 | 0.0209104 | 0.0036 | 4.00E-09 | -0.0104 | 0.0194 | 0.5908 |
| rs12900061 | 15 | 6.60E+07 | A | G | 0.1623 | 0.0365775 | 0.0059 | 4.80E-10 | -0.0069 | 0.0207 | 0.7383 |
| rs28420834 | 15 | 8.30E+07 | G | A | 0.4291 | 0.0289136 | 0.0045 | 1.20E-10 | -0.0296 | 0.0168 | 0.07839 |

SNP – single nucleotide polymorphism; CHR – chromosome’ POS- position; A1 – effect allele 1; A2 – other allele 2; EAF – effect allele frequency; BETA - beta coefficient for association between each SNP and intelligence; SE – standard error for associations with each SNP; P – P value for associations with each SNP; LOR – Log odds ratios for the association between each SNP Alzheimer’s disease

**Table S3: Total and direct effects of years of schooling and intelligence on risk of Alzheimer’s disease: A comparison of effect estimates from inverse variance weighted and MR-Egger regression.**

|  |  | **Causal effect estimates** | |  | **Heterogeneity statistics** | | |
| --- | --- | --- | --- | --- | --- | --- | --- |
| **Total effects** | **N SNPs** | **OR (95% CI)** | **P** |  | **Q** | **df** | **P** |
| **Years of schooling** |  |  |  |  |  |  |  |
| Inverse variance weighted estimate | 142 | 0.62 (0.51, 0.77) | 7.25E-06 |  | 179.33 | 141 | 0.02 |
| MR Egger estimate |  | 0.40 (0.15, 1.02) | 0.06 |  | 178.05 | 140 | 0.02 |
| MR Egger intercept |  | 0.008 (-0.01, 0.02) | 0.33 |  | - | - | - |
|  |  |  |  |  |  |  |  |
| **Intelligence** |  |  |  |  |  |  |  |
| Inverse variance weighted estimate | 185 | 0.65 (0.57, 0.75) | 1.13E-10 |  | 226.43 | 184 | 0.02 |
| MR Egger estimate |  | 1.36 (0.75, 2.48) | 0.31 |  | 218.95 | 183 | 0.04 |
| MR Egger intercept |  | -0.02 (-0.03, -0.004) | 0.01 |  | - | - | - |
| **Independent effects** |  | **OR (95% CI)** | **P** |  |  |  |  |
| **Years of schooling** |  |  |  |  |  |  |  |
| Inverse variance weighted estimate | 231 | 1.15 (0.68, 1.93) | 0.60 |  | - | - | - |
| MR Egger estimate |  | 1.36 (0.75, 2.48) | 0.13 |  | - | - | - |
| MR Egger intercept* |  | -0.01 (-0.02, -.0002) | 0.06 |  | - | - | - |
|  |  |  |  |  |  |  |  |
| **Intelligence** |  |  |  |  |  |  |  |
| Inverse variance weighted estimate | 231 | 0.62 (0.44, 0.88) | 0.008 |  | - | - | - |
| MR Egger estimate |  | 0.85 (0.53, 1.38) | 0.52 |  | - | - | - |
| MR Egger intercept* |  | -0.01 (-0.02, -.0002) | 0.06 |  | - | - | - |

*Note that the intercept estimates are the same as there is only one intercept in the multivariable MR model (i.e. for independent effects of intelligence and education on AD risk)

**Table S4: Sensitivity analysis for the bidirectional effect of intelligence on years of schooling and vice versa, excluding SNPs for education for which there was evidence that the hypothesised causal direction was incorrect (i.e. there was evidence the SNPs explained more variation in intelligence than education)**

| **Total effects** | **N SNPs** | **Standardised** **β  (95% CI)** | **P** |
| --- | --- | --- | --- |
| Years of schooling on intelligence | 23 | 0.57 (0.48, 0.66) | 1.73E-36 |

SNP – single nucleotide polymorphism. CI – confidence interval. Results are interpreted per one standard deviation (3.6 years) increase in years of schooling

**Table S5: Bidirectional effect of intelligence on years of schooling and vice versa: A comparison of effect estimates from inverse variance weighted and MR-Egger regression.**

|  |  | **Causal effect estimates** | |  | **Heterogeneity statistics** | | |
| --- | --- | --- | --- | --- | --- | --- | --- |
|  | **N SNPs** | **B (95% CI)** | **P** |  | **Q** | **df** | **P** |
| **Intelligence on years of schooling** |  |  |  |  |  |  |  |
| Inverse variance weighted estimate | 180 | 0.51 (0.49, 0.54) | 1.77E-95 |  | 318.22 | 179 | <0.001 |
| MR Egger estimate |  | 0.57 (0.46, 0.68) | 2.77E-20 |  | 316.34 | 178 | <0.001 |
| MR Egger intercept |  | -0.001 (-0.004, 0.001) | 0.32 |  | - | - | - |
|  |  |  |  |  |  |  |  |
| **Years of schooling on intelligence** |  |  |  |  |  |  |  |
| Inverse variance weighted estimate | 148 | 1.04 (0.99, 1.10) | 9.36E-80 |  | 330.31 | 147 | <0.001 |
| MR Egger estimate |  | 1.04 (0.83, 1.25) | 1.17E-18 |  | 330.31 | 146 | <0.001 |
| MR Egger intercept |  | 0.00001 (-0.004, 0.004) | 0.99 |  | - | - | - |


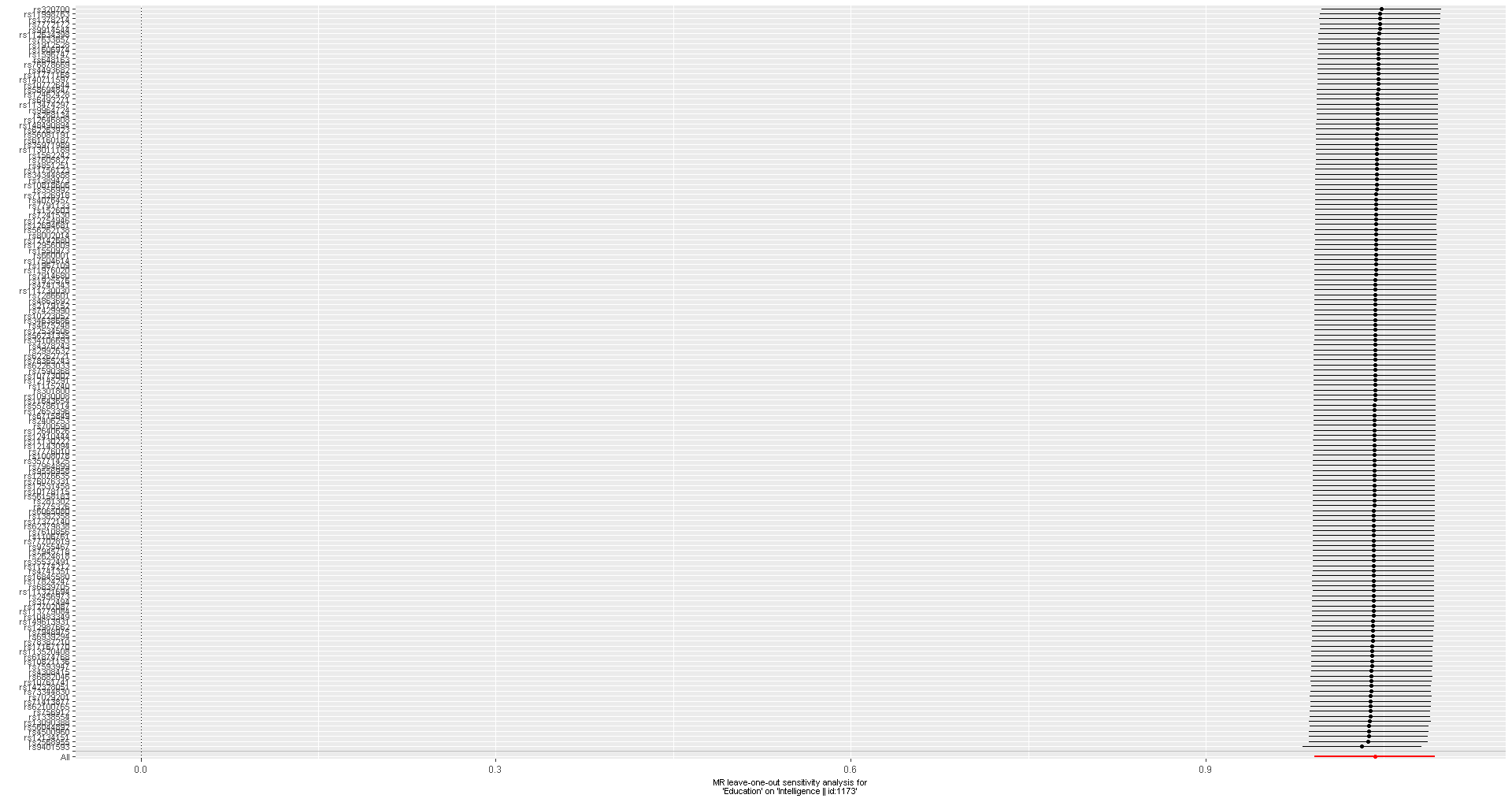

**Figure S1: Leave-one-out plot for univariable analysis of years of schooling on intelligence**

**
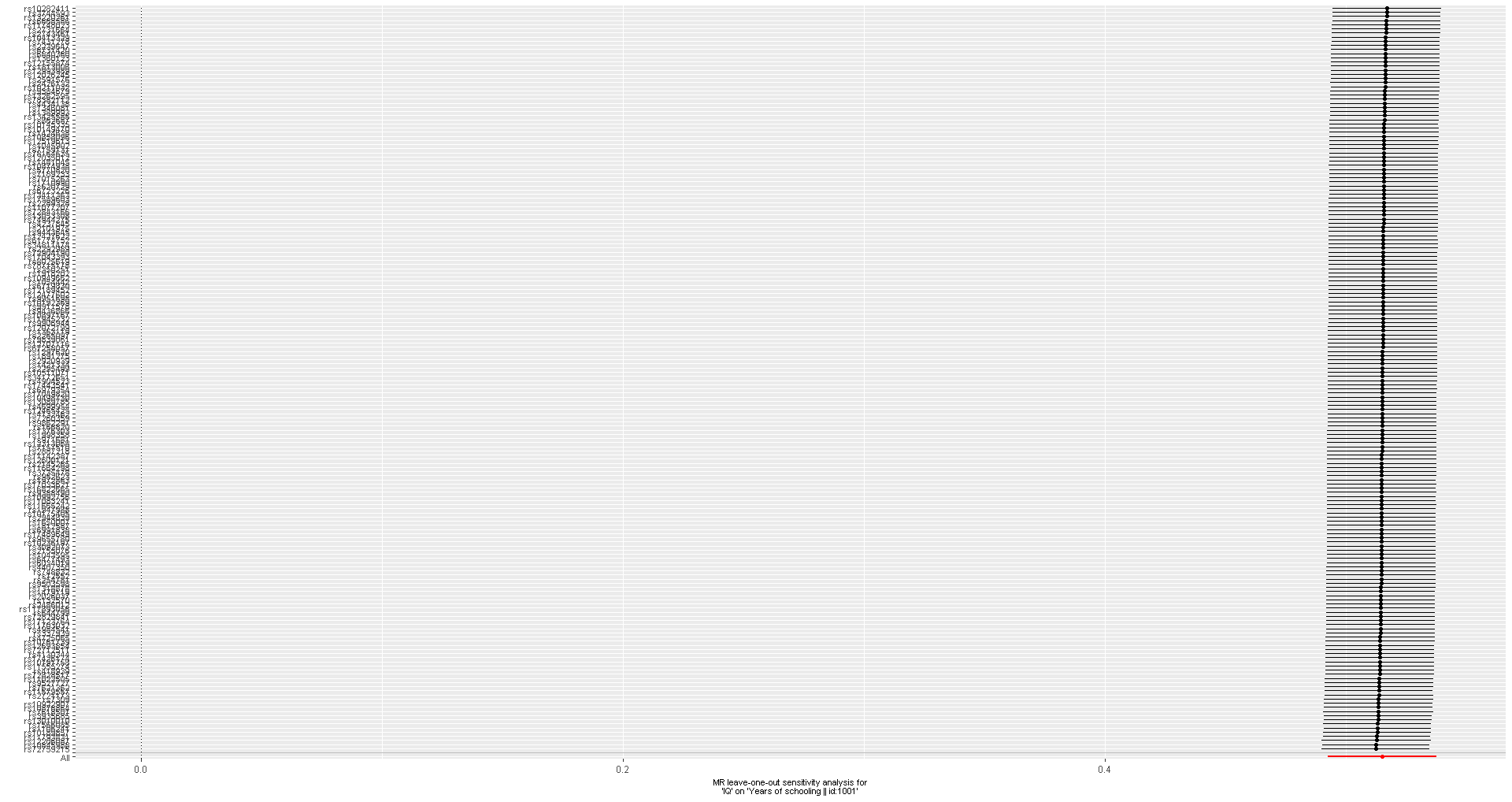
**

**Figure S2: Leave-one-out plot for univariable analysis of intelligence on years of schooling**

**
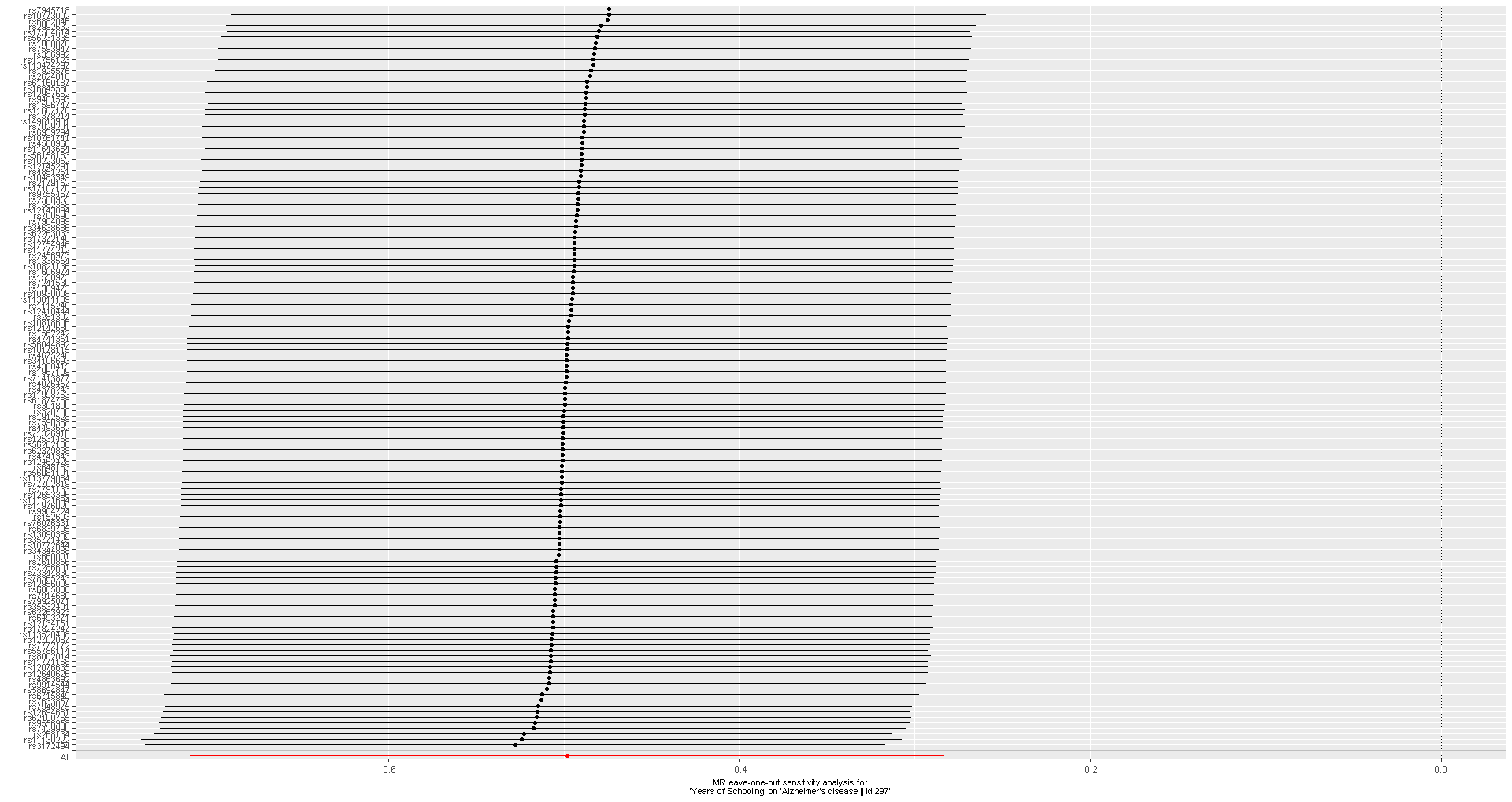
Figure S3: Leave-one-out plot for univariable analysis of years of schooling on AD risk**

**
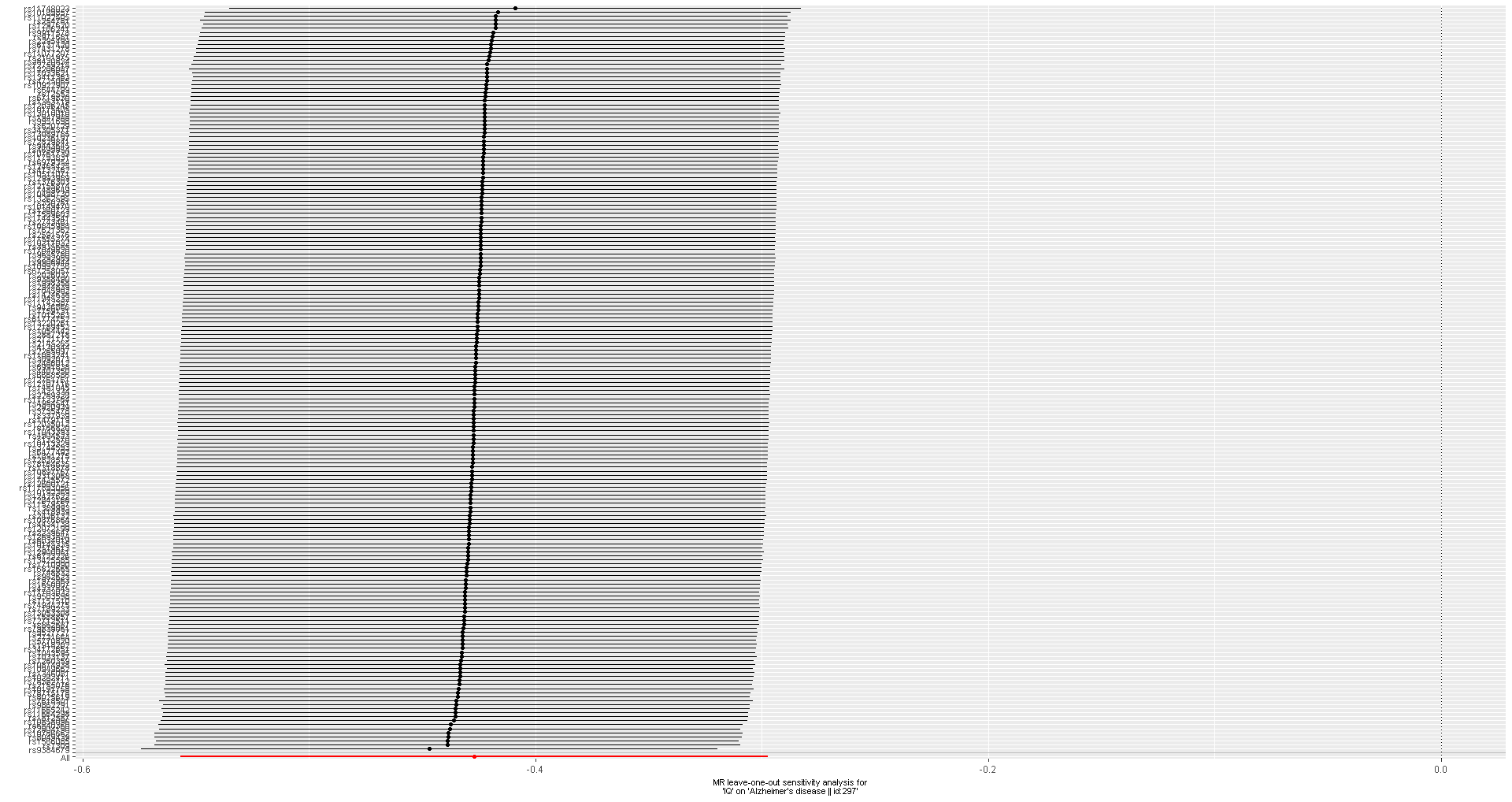
Figure S4: Leave-one-out plot for univariable analysis of years of schooling on AD risk**

**
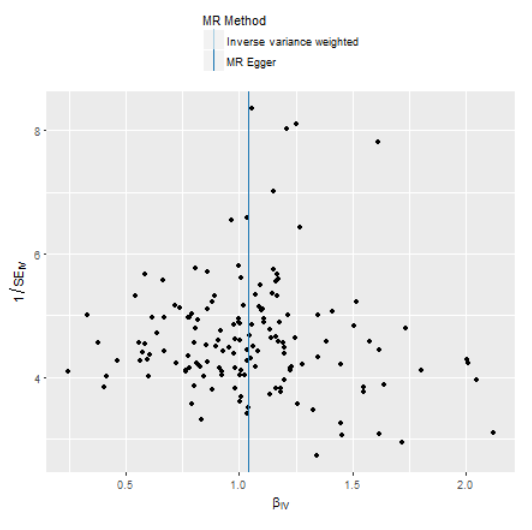

Figure S5: Funnel plot assessing the extent to which pleiotropy is balanced across the set of instruments used in the univariable MR analysis of years of schooling on intelligence**

**
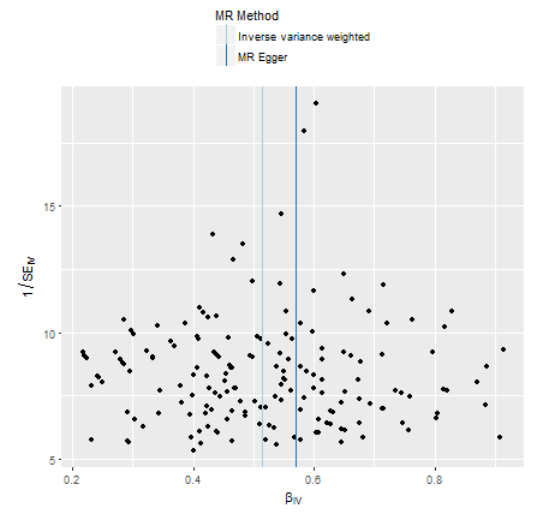

Figure S6: Funnel plot assessing the extent to which pleiotropy is balanced across the set of instruments used in the univariable MR analysis of intelligence on years of schooling**

**
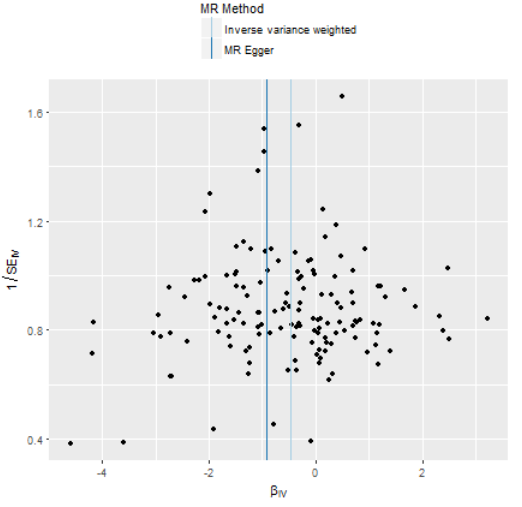
**
**Figure S7: Funnel plot assessing** **the extent to which pleiotropy is balanced across the set of instruments used in the univariable MR analysis of years of schooling on risk of AD**

**
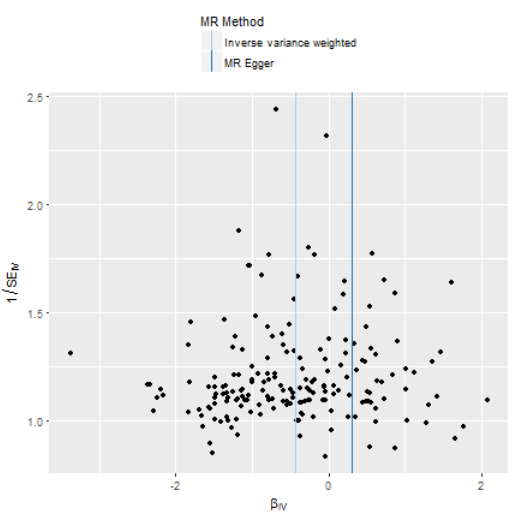

Figure S8: Funnel plot assessing the extent to which pleiotropy is balanced across the set of instruments used in the univariable MR analysis of intelligence on risk of AD**

**References**

1. Okbay A, Beauchamp JP, Fontana MA, et al. Genome-wide association study identifies 74 loci associated with educational attainment. *Nature.* 2016;533(7604):539-542.

2. Bowden J, Davey Smith G, Burgess S. Mendelian randomization with invalid instruments: effect estimation and bias detection through Egger regression. *Int J Epidemiol.* 2015;44(2):512-525.

3. Haycock PC, Burgess S, Wade KH, Bowden J, Relton C, Davey Smith G. Best (but oft-forgotten) practices: the design, analysis, and interpretation of Mendelian randomization studies. *Am J Clin Nutr.* 2016;103(4):965-978.

4. Bowden J, Del Greco MF, Minelli C, Davey Smith G, Sheehan N, Thompson J. A framework for the investigation of pleiotropy in two-sample summary data Mendelian randomization. *Stat Med.* 2017.
